# Supplementary material for: Perioperative Vascular Biomarker Profiling in Elective Surgery Patients Developing Postoperative Delirium: A Prospective Cohort Study
Source: Biomedicines. 2021 May 15;9(5):553. doi: 10.3390/biomedicines9050553 (PMC8155907; doi:10.3390/biomedicines9050553)
Supplement: Supplementary file 1 [file biomedicines-09-00553-s001.zip › S1.pdf]

Supplemental Table S1: Pre- and postoperative serum biomarker profiling

	Preoperative (pg/mL)	Postoperative (pg/mL)	*p value*
Vascular activation/ permeability:			
E-Selectin	29945 (20522 - 43614)	21461 (14886 - 34171)	***<0.0001***
ICAM-1	224783 (166416 - 381149)	209646 (153892 - 338841)	***<0.0001***
VCAM-1	1121475 (853534 - 1727897)	1439545 (1097545 - 2050708)	***<0.0001***
SDC1	1910 (1477 - 2818)	2662 (1808 - 3958)	***<0.0001***
THBD	6878 (5302 - 8262)	6336 (5217 - 7795)	***0.0064***
ANGPT2	2115 (1532 - 3196)	2657 (1735 - 3730)	***0.0002***
TIE2	13859 (10919 - 17791)	9748 (6672 - 13381)	***<0.0001***
Inflammation:			
IL-8	12.05 (5.28 - 23.16)	24.48 (13.16 - 39.51)	***<0.0001***
CCL2	324.5 (254.3 - 439.1)	435.8 (277.2 - 779.8)	***<0.0001***
RAGE	2244 (1481 - 3077)	2641 (1838 - 3561)	***0.0072***
Resistin	16375 (11268 - 23379)	24835 (18029 - 35389)	***<0.0001***
CXCL5	673.0 (248.9 - 1062)	523.6 (232.1 - 919.8)	***0.0008***
uPAR	215.3 (109.3 - 350.3)	250 (11.3 - 400.1)	*0.1469*
NSE	18758 (11204 - 29300)	25204 (17293 - 45056)	***<0.0001***

Data are given as median values with 25th and 75th percentile and were compared using Wilcoxon rank-sum test. n = 118

ICAM-1 = Intercellular Adhesion Molecule 1, VCAM-1 = Vascular Cell Adhesion Protein 1, SDC1 = Syndecan-1, THBD = Thrombomodulin, ANGPT2 = Angiopoietin-2, TIE2 = Tyrosine Kinase with Immunoglobulin-like and EGF-like domains 2, IL-8 = Interleukin-8, CCL2 = CC-chemokine Ligand 2, RAGE = Receptor for Advanced Glycation Endproducts, CXCL5 = C-X-C Motif Chemokine 5, uPAR = Urokinase Plasminogen Activator Surface Receptor, NSE = Neuron-specific Enolase
