# Supplementary material for: Perioperative Vascular Biomarker Profiling in Elective Surgery Patients Developing Postoperative Delirium: A Prospective Cohort Study
Source: Biomedicines. 2021 May 15;9(5):553. doi: 10.3390/biomedicines9050553 (PMC8155907; doi:10.3390/biomedicines9050553)
Supplement: Supplementary file 1 [file biomedicines-09-00553-s001.zip › S2.pdf]

Supplemental Table S2: Patient and procedural details (no-POD and POD)

Parameter	no-POD	POD	*p value*
Patient details:			
n (%)	85 (72)	33 (28)	
Age (years)	71 (66 - 77)	73 (68 - 80)	*0.2782*
Male gender (n [%])	42 (49)	28 (85)	***0.0004***
Body mass index (kg/m^2)	27.7 (24.2 - 31.2)	26.3 (24.5 - 28.9)	*0.1896*
ASA status:			
I (n [%])	2 (2.4)	1 (3.0)	*>0.9999*
II (n [%])	30 (35.3)	5 (15.1)	***0.0425***
III (n [%])	45 (52.9)	25 (75.8)	***0.0359***
IV (n [%])	8 (9.4)	2 (6.1)	*0.7237*
Preop. routine laboratory values:			
Hemoglobin (g/dl)	13.4 (11.9 - 14.5)	13.4 (12.4 - 14.5)	*0.9845*
HbA_{1C} (%)	5.6 (5.4 - 6.0)	5.7 (5.3 - 6.5)	*0.3937*
Leukocyte count (G/l)	7.1 (6.0 - 9.0)	6.7 (5.7 - 7.6)	*0.1916*
Sodium (mmol/l)	140 (138 - 142)	141 (139 - 142)	*0.0732*
Potassium (mmol/l)	4.5 (4.1 - 4.8)	4.3 (4.0 - 4.6)	*0.1116*
Creatinine (mg/dl)	0.9 (0.79 - 1.05)	0.9 (0.77 - 1.09)	*0.8010*
C-reactive protein (mg/l)	2.9 (1.1 - 7.4)	4.2 (1.3 - 14.0)	*0.4384*
Total protein (g/l)	70 (65 - 73)	68 (65 - 74)	*0.8656*
HS cardiac troponin T (ng/l)	12.0 (7.5 - 19.1)	14.5 (8.9 - 21.9)	*0.1896*
NT-proBNP (pg/ml)	241 (108 - 648)	228 (141 - 1520)	*0.3855*
Procedural and anesthesia details:			
Surgical risk:			
Low (n [%])	10 (11.8)	1 (3.0)	*0.1788*
Intermediate (n [%])	36 (42.3)	11 (33.3)	*0.4085*
High (n [%])	39 (45.9)	21 (63.7)	*0.1023*
Surgical specialty:			
General (n [%])	14 (17)	4 (12)	*0.7763*
Orthopedic and trauma (n [%])	31 (37)	4 (12)	***0.0126***
Cardiac (n [%])	19 (22)	18 (55)	***0.0016***
Thoracic (n [%])	2 (2)	0 (0)	*>0.9999*
Vascular (n [%])	1 (1)	3 (9)	*0.0658*
Ear-nose-throat (n [%])	9 (11)	2 (6)	*0.7256*
Urologic (n [%])	7 (8)	2 (6)	*>0.9999*
Plastic (n [%])	2 (2)	0 (0)	*>0.9999*
Placement of epidural catheter (n [%])			
Duration of surgery (min)	204 (132 - 278)	249 (168 - 336)	*0.1324*
Duration of mechanical ventilation (h)	5.1 (3.4 - 10.8)	11.0 (4.6 - 26.1)	***0.0017***
Postoperative admission to ICU (n [%])	41 (48)	25 (76)	***0.0076***
Length of hospital stay (days)	13 (9 - 20)	14 (10 - 28)	*0.2015*

Data are given as percentage values (of subcohort) or as median values with 25th and 75th percentile, respectively. Data were compared using Fisher's exact test or Mann-Whitney U test, respectively. ASA = American Society of Anaesthesiologists, HS cardiac troponin T = High-sensitive cardiac troponin T, NT-proBNP = N-terminal prohormone of brain natriuretic peptide, ICU = Intensive Care Unit
