# Supplementary material for: Perioperative Vascular Biomarker Profiling in Elective Surgery Patients Developing Postoperative Delirium: A Prospective Cohort Study
Source: Biomedicines. 2021 May 15;9(5):553. doi: 10.3390/biomedicines9050553 (PMC8155907; doi:10.3390/biomedicines9050553)
Supplement: Supplementary file 1 [file biomedicines-09-00553-s001.zip › S3.pdf]

Supplemental Table S3: Pre- and postoperative serum biomarker profiling (no-POD and POD)

	Preoperative			***Postoperative***		
	no-POD (n = 85)	POD (n = 33)	*p value*	no-POD (n = 85)	POD (n = 33)	*p value*
Vascular activation/ permeability:						
E-Selectin	27238 (19896 - 37631)	36796 (22719 - 52220)	*0.06*	20743 (14505 - 33214)	26411 (16729 - 35875)	*0.17*
ICAM-1	219620 (170122 - 388059)	233475 (160310 - 387719)	*0.97*	208526 (157019 - 339071)	221653 (139500 - 333811)	*0.71*
VCAM-1	1038317 (846982 - 1671950)	1257490 (872532 - 1816731)	*0.27*	1422086 (1097336 - 1888507)	1727963 (1083363 - 2326175)	*0.23*
SDC1	2006 (1405 - 2767)	1891 (1556 - 3343)	*0.71*	2451 (1706 - 3836)	3139 (2072 - 4102)	*0.07*
THBD	6453 (5246 - 8084)	7168 (5820 - 9508)	*0.10*	6228 (5108 - 7677)	6642 (5297 - 8338)	*0.36*
ANGPT2	2224 (1594 - 3198)	1883 (1432 - 3192)	*0.45*	2730 (1813 - 3816)	2368 (1362 - 3573)	*0.29*
TIE2	13791 (10916 - 17138)	14078 (10913 - 19649)	*0.63*	10263 (6775 - 14018)	7970 (5245 - 11794)	*0.07*
Inflammation:						
IL-8	11.77 (3.11 - 24.38)	13.98 (7.90 - 22.48)	*0.51*	24.08 (12.81 - 35.55)	33.15 (17.32 - 49.95)	*0.08*
CCL2	324.8 (256.0 - 433.2)	324.3 (253.3 - 456.7)	*0.97*	385.2 (271.2 - 635.7)	675.6 (286.1 - 1600)	***0.03***
RAGE	2256 (1438 - 2948)	2159 (1503 - 3624)	*0.32*	2424 (1786 - 3457)	2789 (1973 - 3685)	*0.27*
Resistin	15992 (11114 - 23467)	18374 (12754 - 23707)	*0.76*	26647 (18165 - 35119)	23530 (17638 - 37011)	*0.98*
CXCL5	700.2 (301.5 - 1076)	594.3 (171.0 - 954.4)	*0.48*	584.3 (224.1 - 984.8)	347.1 (231.6 - 892.7)	*0.31*
uPAR	222.1 (114.9 - 350.7)	197.8 (78.2 - 373.7)	*0.54*	263.8 (137.4 - 425.4)	207.1 (96.56 - 382.6)	*0.28*
NSE	19986 (11736 - 30321)	15875 (8262 - 25806)	*0.15*	25289 (18995 - 45945)	23317 (14812 - 43536)	*0.39*

Data are given as median values with 25th and 75th percentile and were compared using Mann-Whitney U test.

ICAM-1 = Intercellular Adhesion Molecule 1, VCAM-1 = Vascular Cell Adhesion Protein 1, SDC1 = Syndecan-1, THBD = Thrombomodulin, ANGPT2 = Angiopoietin-2, TIE2 = Tyrosine Kinase with Immunoglobulin-like and EGF-like domains 2, IL-8 = Interleukin-8, CCL2 = CC-chemokine Ligand 2, RAGE = Receptor for Advanced Glycation Endproducts, CXCL5 = C-X-C Motif Chemokine 5, uPAR = Urokinase Plasminogen Activator Surface Receptor, NSE = Neuron-specific Enolase
