# Supplementary material for: Perioperative Vascular Biomarker Profiling in Elective Surgery Patients Developing Postoperative Delirium: A Prospective Cohort Study
Source: Biomedicines. 2021 May 15;9(5):553. doi: 10.3390/biomedicines9050553 (PMC8155907; doi:10.3390/biomedicines9050553)
Supplement: Supplementary file 1 [file biomedicines-09-00553-s001.zip › S4.pdf]

Supplemental Table S4: Pre- and postoperative serum biomarker profiling (no-POD and POD) following propensity score matching

	Preoperative			***Postoperative***		
	no-POD (n = 33)	POD (n = 33)	*p value*	no-POD (n = 33)	POD (n = 33)	*p value*
Vascular activation/ permeability:						
E-Selectin	30316 (17996 - 37610)	36796 (22619 - 52220)	*0.16*	21597 (14148 - 32771)	26411 (16729 - 35875)	*0.35*
ICAM-1	213139 (164919 - 532964)	233475 (160310 - 387719)	*0.87*	206570 (152350 - 389457)	221653 (139500 - 333811)	*0.99*
VCAM-1	1178782 (959076 - 1914238)	1257490 (872532 - 1816731)	*0.34*	1598081 (1304911 - 2058955)	1727963 (1083363 - 2326175)	*0.61*
SDC1	2387 (1458 - 3703)	1891 (1556 - 3343)	*0.47*	2783 (2260 - 4037)	3139 (2072 - 4102)	*0.52*
THBD	7411 (5349 - 8353)	7168 (5820 - 9508)	*0.32*	6393 (5550 - 7761)	6642 (5297 - 8338)	*0.55*
ANGPT2	2058 (1393 - 3312)	1883 (1432 - 3192)	*0.51*	2297 (1400 - 3980)	2368 (1362 - 3573)	*0.61*
TIE2	12521 (10659 - 16925)	14078 (10913 - 19649)	*0.24*	7803 (5540 - 10822)	7970 (5245 - 11794)	*0.62*
Inflammation:						
IL-8	15.43 (9.44 - 30.08)	13.98 (7.895 - 22.48)	*0.24*	25.72 (15.55 - 50.26)	33.15 (17.32 - 49.95)	*0.51*
CCL2	312.4 (268.3 - 403.8)	324.3 (253.3 - 456.7)	*0.48*	366.7 (267.5 - 744.8)	675.6 (286.1 - 1600.0)	***0.01***
RAGE	2398 (1666 - 3198)	2159 (1503 - 3624)	*0.53*	2568 (1934 - 3515)	2789 (1973 - 3685)	*0.23*
Resistin	14548 (10545 - 22408)	18374 (12754 - 23707)	*0.54*	22724 (16912 - 35638)	23530 (17638 - 37011)	*0.70*
CXCL5	704.1 (331.0 - 1184.0)	594.3 (171.0 - 954.4)	*0.34*	560.9 (263.4 - 810.2)	347.1 (231.6 - 892.7)	*0.71*
uPAR	199.2 (103.5 - 335.4)	197.8 (78.21 - 373.7)	*0.93*	258.3 (141.9 - 336.5)	207.1 (96.56 - 382.6)	*0.74*
NSE	16860 (11597 - 23030)	15875 (8262 - 25806)	*0.76*	20092 (16269 - 24845)	23317 (14812 - 43536)	*0.26*

Data are given as median values with 25th and 75th percentile and were compared using Wilcoxon rank-sum test.

ICAM-1 = Intercellular Adhesion Molecule 1, VCAM-1 = Vascular Cell Adhesion Protein 1, SDC1 = Syndecan-1, THBD = Thrombomodulin, ANGPT2 = Angiopoietin-2, TIE2 = Tyrosine Kinase with Immunoglobulin-like and EGF-like domains 2, IL-8 = Interleukin-8, CCL2 = CC-chemokine Ligand 2, RAGE = Receptor for Advanced Glycation Endproducts, CXCL5 = C-X-C Motif Chemokine 5, uPAR = Urokinase Plasminogen Activator Surface Receptor, NSE = Neuron-specific Enolase
